# Cytokine concentration in peripheral blood of patients with colorectal cancer

**DOI:** 10.3389/fimmu.2023.1175513

**Published:** 2023-03-30

**Authors:** Wenchang Li, Fangqian Chen, Han Gao, Zhuoqing Xu, Yu Zhou, Shenjie Wang, Zeping Lv, Yuchen Zhang, Zifeng Xu, Jianting Huo, Jingkun Zhao, Yaping Zong, Wenqing Feng, Xiaohui Shen, Zhiyuan Wu, Aiguo Lu

**Affiliations:** ^1^Department of General Surgery, Ruijin Hospital, Shanghai Jiaotong University School of Medicine, Shanghai, China; ^2^Department of Radiology, Ruijin Hospital, Shanghai Jiaotong University School of Medicine, Shanghai, China; ^3^Shanghai Institute of Digestive Surgery, Ruijin Hospital, Shanghai Jiao Tong University School of Medicine, Shanghai, China; ^4^Shanghai Minimally Invasive Surgery Center, Ruijin hospital, Shanghai Jiaotong University School of Medicine, Shanghai, China

**Keywords:** colorectal cancer, tumor immune microenvironment, peripheral blood, cytokines, Luminex

## Abstract

**Introduction:**

The role of tumour secretory cytokines and peripheral circulatory cytokines in tumour progression has received increasing attention; however, the role of tumour-related inflammatory cytokines in colorectal cancer (CRC) remains unclear. In this study, the concentrations of various cytokines in the peripheral blood of healthy controls and patients with CRC at different stages were compared.

**Methods:**

Peripheral blood samples from 4 healthy participants and 22 colorectal cancer patients were examined. Luminex beads were used to evaluate concentration levels of 40 inflammatory cytokines in peripheral blood samples.

**Results:**

In peripheral blood, compared with healthy controls and early stage (I + II) CRC patients, advanced CRC (III + IV) patients had increased concentrations of mononuclear/macrophage chemotactic-related proteins (CCL7, CCL8, CCL15, CCL2, and MIF), M2 polarization-related factors (IL-1β, IL-4), neutrophil chemotactic and N2 polarization-related cytokines (CXCL2, CXCL5, CXCL6, IL-8), dendritic cells (DCs) chemotactic-related proteins (CCL19, CCL20, and CCL21), Natural killer (NK) cell related cytokines (CXCL9, CXCL10), Th2 cell-related cytokines (CCL1, CCL11, CCL26), CXCL12, IL-2, CCL25, and CCL27, and decreased IFN-γ and CX3CL1 concentrations. The differential upregulation of cytokines in peripheral blood was mainly concentrated in CRC patients with distant metastasis and was related to the size of the primary tumour; however, there was no significant correlation between cytokine levels in peripheral blood and the propensity and mechanism of lymph node metastasis.

**Discussion:**

Different types of immune cells may share the same chemokine receptors and can co-localise in response to the same chemokines and exert synergistic pro-tumour or anti-tumour functions in the tumour microenvironment. Chemokines and cytokines affect tumour metastasis and prognosis and may be potential targets for treatment.

## 1 Introduction

Colorectal cancer (CRC) is one of the most common malignant tumors of the digestive tract and the third most common cancer in the world (1). Inflammation can promote cancer tumorigenesis and progression and plays a role in all steps of tumor cell transformation, proliferation, invasion, and metastasis (2–4). Although the mechanisms by which chronic inflammation promotes tumor progression remain elusive, the accumulation of inflammatory cells and inflammatory factors in the tumor microenvironment (TME) has been found to promote angiogenesis, malignant cell proliferation and metastasis, and epithelial-mesenchymal transition (EMT), and reverse the acquired immune response. Additionally, it alters the sensitivity of tumor cells to hormones and chemotherapeutic agents (5–7).

During an inflammatory response, an extremely complex regulatory network involves pro-inflammatory cytokines, pro-inflammatory cytokine-releasing cells, and pro-inflammatory cytokine target cells (8). In addition to pro-inflammatory cytokines, there are many other inflammatory mediators, which are small molecular compounds closely associated with vascular, nervous system, and cellular proliferative responses (9). The immunological TME plays a vital role in the development of CRC (10, 11), in which cancer cells communicate with neighboring cells through soluble factors, such as cytokines or chemokines, to produce a favorable TME (12). In addition, systemic chronic inflammation (obesity, depression, and so on) and treatment-induced chronic inflammation promote tumorigenesis and progression by affecting the immune system (11).

At different stages of CRC, different inflammatory cytokines and cells are involved, each playing its own role. In the early stages of CRC, disruption of the intestinal epithelial barrier by bacterial infection, microbial metabolites, obesity, or epithelial injury leads to the production and release of several proinflammatory cytokines. However, alterations in the gut microbiota can promote tumorigenesis because the microbiota and intestinal epithelial cells interact in a complex network to maintain homeostasis (13). Disruption of the intestinal barrier at the colorectal tumor site induces activation of innate immune cells and increased expression of pro-inflammatory cytokines such as tumor necrosis factor (TNF), one of the earliest and most important pro-inflammatory cytokines that activates other pro-inflammatory cytokines through the nuclear factor kappa-B (NF-κB) signaling pathway (14). During tumor progression, chemokines attract more immune cells to the tumor.

Most studies on markers of circulating inflammation have focused on a small number of candidate markers (15–17); nonetheless, these markers represent only a small part of the inflammatory cascade. Inflammatory processes are complex responses to stimuli and involve the interaction of host cells and signaling molecules (i.e. pro-inflammatory and anti-inflammatory cytokines, growth factors, angiogenic factors, and chemokines) (18). Few studies have comprehensively measured the relationship between these circulating cytokines and the development and progression of colorectal cancer. Therefore, in this study, we conducted a case-control study to compare the levels of 40 serum cytokines, including pro-inflammatory and anti-inflammatory cytokines, chemokines, growth factors, and angiogenic factors, in patients with colorectal cancer versus non-tumor patients, and to assess changes in cytokine levels at different CRC stages.

## 2 Materials and methods

### 2.1 Patients and specimens

This study was approved by the Biomedical Ethics Committee of the Ruijin Hospital. Between 2021 and 2022, peripheral blood samples were collected from 22 CRC patients and 4 non-tumor patients at the Shanghai Minimally Invasive Surgery Center of Ruijin Hospital for Luminex assay (**Table S1**). All CRC patients were pathologically diagnosed with colorectal cancer and underwent laparoscopic surgery at our center. The inclusion criteria were as follows: patients diagnosed with CRC who were candidates for surgery and those who were not receiving neoadjuvant therapy. The exclusion criteria were inflammatory disease, autoimmune disease, infection, immunosuppression, or immunoregulatory therapy. Tissue sections from each patient were TNM staged according to the 2015 National Comprehensive Cancer Network (NCCN) guidelines. All patients signed an informed consent form and were fully informed about the study. Peripheral blood (5 mL) was collected from each participant using standard sterilization procedures, and serum components were centrifuged and immediately stored in a freezer at -80°C until further analysis. Clinical data of the patients are summarized in **Table S1**.

### 2.2 Luminex assay

As previously described (19), cytokines were measured using a Luminex X-MAP system (Luminex 200 system, Luminex Corporation, Austin, TX, USA) and the Bio-Plex Pro Human Chemokine Panel 40-plex (Bio-Rad Laboratories, California, USA). The assay was conducted according to the manufacturer’s protocol (Wayen Biotechnologies, Shanghai, China). The Bio-Plex Pro Human Chemokine Panel 40-plex allows the simultaneous detection of the following circulating analytes: 6Ckine/CCL21, BCA-1/CXCL13, CTACK/CCL27, ENA-78/CXCL5, Eotaxin-2/CCL24, Eotaxin-3/CCL26, Eotaxin/CCL11, Fractalkine/CX3CL1, GCP-2/CXCL6, GM-CSF, Gro-α/CXCL1, Gro-β/CXCL2, I-309/CCL1, I-TAC/CXCL11, IFN-γ, IL-10, IL-16, IL-1β, IL-2, IL-4, IL-6, IL-8/CXCL8, IP-10/CXCL10, MCP-1/CCL2, MCP-2/CCL8, MCP-3/CCL7, MCP-4/CCL13, MDC/CCL22, MIF, MIG/CXCL9, MIP-1α/CCL3, MIP-1δ/CCL15, MIP-3α/CCL20, MIP-3b/CCL19, MPIF-1/CCL23, SCYB16/CXCL16, SDF-1α+β/CXCL12, TARC/CCL17, TECK/CCL25, and TNF-α. Additional information on cytokines is summarized in **Table S2**. Concentrated human recombinant standards were provided by the vendor and a broad range of standards was used to establish standard curves. The samples and standards tested in this experiment were detected using a Luminex 200 detector, and the fluorescence obtained was automatically calculated and optimized using the software. The original fluorescence of each sample was substituted into the standard curve formula to calculate sample concentration.

### 2.3 Cell lines

The human CRC cell lines were purchased from the American Type Culture Collection (ATCC, USA). All cells were cultured in RPMI-1640 medium containing 10% fetal bovine serum (FBS) and 1% penicillin-streptomycin solution and incubated at 37°C and 5% CO_2_.

### 2.4 Cell medium (CM) preparation

CRC cell lines were cultured in RPMI-1640 medium supplemented with 10% fetal bovine serum (FBS) and 1% penicillin-streptomycin. When the cells reached 100% confluence, the supernatant was removed and replaced with fresh medium. The cell medium containing all released cytokines was collected after 24 h.

### 2.5 Statistical analysis

Quantitative data were compared using two-tailed unpaired Student’s t-test or one-way ANOVA. Associations between clinical characteristics were assessed using Pearson’s chi-square test or Fisher’s exact test. Data are shown as mean ± SD. Statistical significance was set at *p* < 0.05. All data were analyzed using SPSS version 20.0 (IBM Corp., Armonk, NY, USA), GraphPad Prism 8.0 (Graph Pad, San Diego, CA, USA), and Microsoft Excel (Microsoft Corporation, Seattle, WA, USA). The drawing materials for the illustrations in this article are from Figdraw (www.figdraw.com).

## 3 Results

### 3.1 Participant characteristics

**Figure 1:**
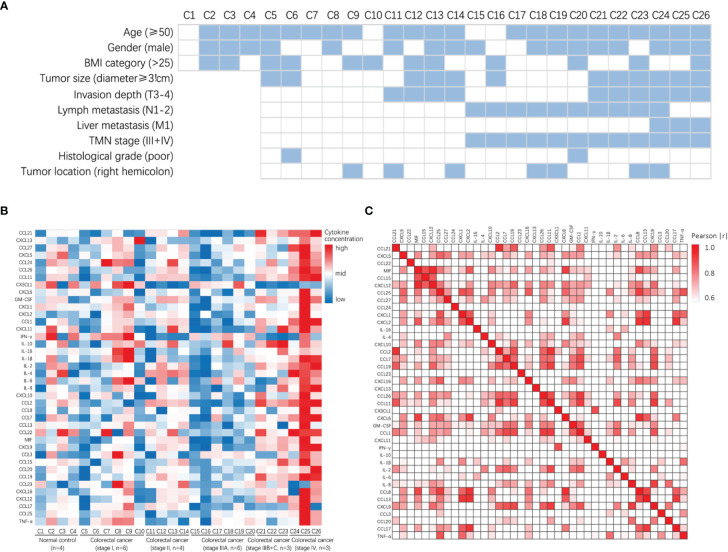
Clinical characteristics and the concentrations of cytokines in patients with colorectal cancer and normal control. **(A)** Heatmap shows clinical characteristics of 26 patients for Luminex assay. **(B)** Heatmap of differentially expressed 40 serum multi-cytokines between participants with colorectal cancer patients and non-tumor patients (healthy controls) using Luminex assay. **(C)** Correlation heatmap of 40 serum cytokines in CRC patients and non-tumor patients (healthy controls).

A total of 26 participants were included in the study, comprising 4 non-tumor patients (healthy controls) and 22 patients with CRC. The heat map shows the clinical information and pathological tumor parameters of the participants (**Figure 1A**). The details of all the participants, including sex and age, are listed in **Table S1**. According to the vendor-recommended program, 40 candidate serum cytokines were selected for evaluation. The heat map shows the concentrations of 40 cytokines in the 26 serum samples (**Figure 1B**). The correlation heatmap showed the correlation between the 40 cytokines (**Figure 1C**).

### 3.2 The distribution of cytokines associated with the monocyte-macrophage system in the peripheral blood of CRC patients

**Figure 2:**
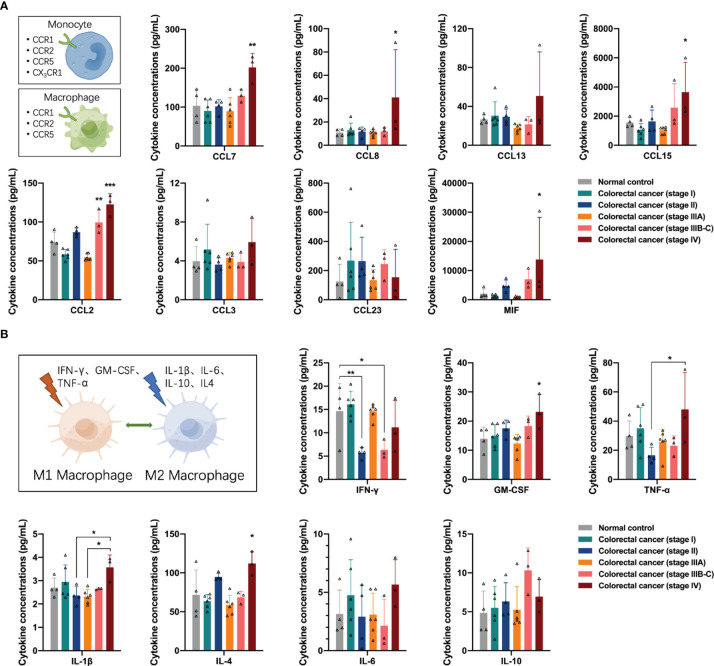
Distribution of cytokines associated with the monocyte-macrophage system in the peripheral blood of the participants. **(A)** Concentrations of CCR1, CCR2, and CCR5 ligands in the peripheral blood of the participants. In colorectal cancer, several monocytes/macrophages are recruited to the tumor region and macrophages are stagnated in the tissues. **(B)** Concentrations of M1 or M2 polarization-related cytokines in the peripheral blood of the participants. M1 macrophage-mediated anti-tumor function was impaired in stage II and stage IIIB-C colorectal cancer and may restore in colorectal cancer patients with distant metastases. M2 macrophage mediated pro-tumor function in patients with stage VI colorectal cancer. Data are presented as mean ± SD, one-way ANOVA, *p < 0.05, **p < 0.01, ***p < 0.001.

First, we analyzed the distribution of cytokines associated with the monocyte-macrophage system in the peripheral blood of participants. Monocytes/macrophages express several CCR1, CCR2, and CCR5 receptors on their surfaces in response to the presence of the corresponding ligands. The concentrations of monocyte chemotaxis-associated proteins CCL7, CCL8, and CCL15 were increased in the peripheral blood of patients with stage IV colorectal cancer (one-way ANOVA, CCL7 control vs stage IV, *p* = 0.0020; CCL8 control vs stage IV, *p* = 0.0336; CCL15 control vs stage IV, *p* = 0.0324); additionally, the concentrations of macrophage chemotaxis-associated protein CCL2 and macrophage migration inhibitory factor (MIF) were increased in the peripheral blood of patients with stage IV colorectal cancer (one-way ANOVA, CCL2 control vs stage IV, *p* < 0.0001, control vs stage IIIB-C, *p* = 0.0088; MIF control vs stage IV, *p* = 0.0191), suggesting that colorectal cancer recruited a large number of monocytes/macrophages to the tumor region and stagnated macrophages in the tissues (**Figure 2A**).

After entering tissues, macrophages can undergo M1 or M2 polarization in response to stimulation by different cytokines in the TME. Many stimuli have been reported in the literature, including IFN-γ, GM-CSF, and TNF-α inducing macrophages to M1 polarization for anti-tumor function, and IL-1β, IL-6, IL-10, and IL-4 inducing macrophages to M2 polarization for pro-tumor function. Next, we analyzed the distribution of these cytokines in the peripheral blood of the participants. Among the factors associated with M1 polarization, the concentrations of IFN-γ in the peripheral blood of patients with stage II and stage IIIB-C colorectal cancer declined (one-way ANOVA, IFN-γ control vs stage II, *p* = 0.0158, control vs stage IIIB-C, *p* = 0.0485), suggesting that the M1 macrophage-mediated anti-tumor function was impaired in these two groups of colorectal cancer patients. In addition, the concentrations of IFN-γ, GM-CSF, and TNF-α in the peripheral blood of patients with stage IV colorectal cancer showed an increasing trend (one-way ANOVA, GM-CSF control vs stage IV, *p* = 0.0176; TNF-α stage II vs stage IV, *p* = 0.0378), suggesting a possible restoration of M1 macrophage-mediated anti-tumor function in colorectal cancer patients with distant metastases. However, the concentrations of M2 polarization-related factors IL-1β and IL-4 also increased in the peripheral blood of patients with stage IV colorectal cancer (one-way ANOVA, IL-1β stage II vs stage IV, *p* = 0.0371; IL-4 control vs stage IV, *p* = 0.0122), suggesting the presence of M2 macrophage-mediated pro-tumor function in patients with stage VI colorectal cancer (**Figure 2B**).

### 3.3 The distribution of neutrophil/G-MDSC-associated cytokines in the peripheral blood of CRC patients

**Figure 3:**
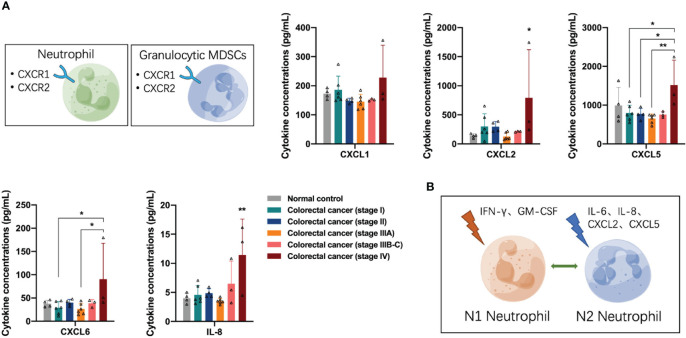
Distribution of neutrophil/granulocyte-myeloid-derived suppressor cell-associated cytokines in the peripheral blood of the participants. **(A)** The concentrations of CXCR1/2 ligands CXCL2, CXCL5, CXCL6, and IL-8 were significantly increased in the peripheral blood of patients with stage IV colorectal cancer. **(B)** Neutrophils undergo N1 or N2 polarization in response to stimulation by different cytokines in the tumor microenvironment. Data are presented as mean ± SD, one-way ANOVA, *p < 0.05, **p < 0.01.

Next, we analyzed the distribution of neutrophil/granulocyte-myeloid-derived suppressor cell (G-MDSC)-associated cytokines in the peripheral blood of participants. Neutrophils and G-MDSCs express several CXCR1 and CXCR2 receptors on the cell surface in response to the binding of the corresponding ligands. The concentrations of CXCR1/2 ligands CXCL2, CXCL5, CXCL6, and IL-8 were increased in the peripheral blood of patients with stage IV colorectal cancer (one-way ANOVA, CXCL2 control vs stage IV, *p* = 0.0306; CXCL5 stage I vs stage IV, *p* = 0.0312; CXCL6 stage I vs stage IV, *p* = 0.0370; IL-8 control vs stage IV, *p* = 0.0038) (**Figure 3A**), suggesting that advanced colorectal cancer recruits a large number of neutrophils to the TME. Similar to macrophages, neutrophils can undergo N1 or N2 polarization in response to stimulation by different cytokines in the TME. Many stimuli have been reported in previous literature, including IFN-γ and GM-CSF, which induce neutrophils to N1 polarization for anti-tumor function, and IL-6, IL-8, CXCL2, and CXCL5, which induce neutrophils to N2 polarization for pro-tumor function (**Figure 3B**). The concentrations of IFN-γ and GM-CSF are shown in **Figure 2B**, the results suggest the impairment of N1 neutrophil-mediated anti-tumor function in patients with stage II and IIIB-C colorectal cancer and recovery in stage IV colorectal cancer. The concentrations of IL-8, CXCL2, and CXCL5 are shown in **Figure 3A**; the results suggest the presence of N2 neutrophil-mediated pro-tumor function in patients with stage IV colorectal cancer.

### 3.4 The distribution of antigen-presenting cell-associated cytokines in the peripheral blood of CRC patients

**Figure 4:**
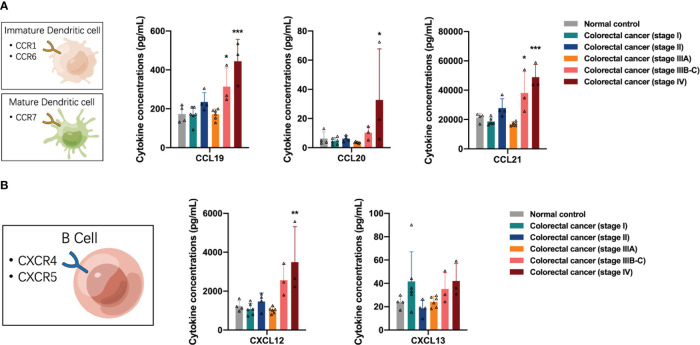
Distribution of antigen-presenting cell-associated cytokines in the peripheral blood of the participants. **(A)** The concentrations of dendritic cells chemotaxis-associated proteins CCL19, CCL20 and CCL21 were elevated in the peripheral blood of patients with stage IIIB-C and IV colorectal cancer. **(B)** The concentrations of CXCL12 were increased in the peripheral blood of patients with advanced colorectal cancer. Data are presented as mean ± SD, one-way ANOVA, *p < 0.05, **p < 0.01, ***p < 0.001.

Next, we examined the distribution of antigen-presenting cell (APC)-associated cytokines in the peripheral blood of participants, including dendritic cells (DCs) and B cells. DCs express many CCR6 and CCR7 receptors on their surface in response to the presence of the corresponding ligands. DCs chemotaxis-associated proteins CCL19, CCL20, and CCL21 were differentially elevated in the peripheral blood of patients with stage IIIB-C and IV colorectal cancer (one-way ANOVA, CCL19 control vs stage IV, *p* < 0.0001; CCL20 control vs stage IV, *p* = 0.0259; CCL21 control vs stage IIIB-C, *p* = 0.0078, control vs stage IV, *p* < 0.0001), suggesting a significant recruitment of DCs, especially mature DCs, in the advanced stage of colorectal cancer (**Figure 4A**). B cells express large numbers of CXCR4 and CXCR5 receptors on their surface in response to CXCL12 and CXCL13, respectively. We found increased concentrations of CXCL12 in the peripheral blood of patients with advanced colorectal cancer (one-way ANOVA, CXCL12 control vs stage IV, *p* = 0.0014), suggesting a tendency for recruitment of B cells in the advanced stage of colorectal cancer (**Figure 4B**).

### 3.5 The distribution of lymphocytes-associated cytokines in the peripheral blood of CRC patients

**Figure 5:**
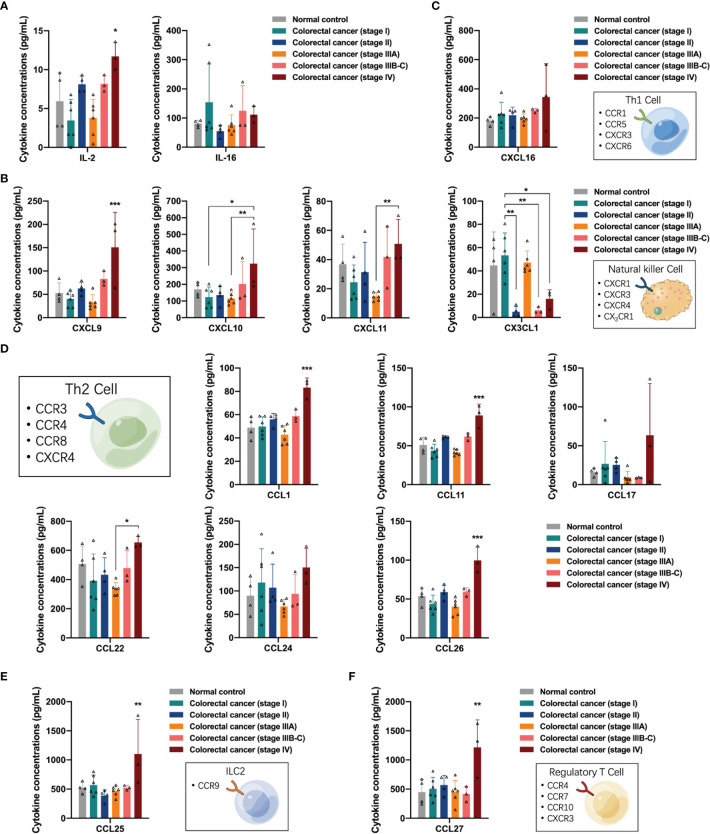
Distribution of lymphocytes-associated cytokines in the peripheral blood of the participants. **(A)** The concentrations of T-cell growth factor IL-2 were significantly increased in peripheral blood of patients with stage IV colorectal cancer. **(B)** The concentrations of CXCL9, CXCL10, and CXCL11 in the peripheral blood of patients with stage IV colorectal cancer were increased, and the concentrations of CX3CL1 in the peripheral blood of patients with stage II, IIIB-C and IV colorectal cancer were decreased. **(C)** Distribution of Th1 cell-related cytokines in the peripheral blood of participants. **(D)** The concentrations of CCL1, CCL11, CCL17, CCL22, and CCL26 were differentially elevated in the peripheral blood of patients with stage IV colorectal cancer. **(E)** Distribution of CCL25 in the peripheral blood of the participants. **(F)** Distribution of CCL27 in the peripheral blood of the participants. Data are presented as mean ± SD, one-way ANOVA, *p < 0.05, **p < 0.01, ***p < 0.001.

Here, we analyzed the regulation of lymphocytes by cytokines in the peripheral blood of participants. First, we observed the distribution of T-cell growth factor IL-2 and T-cell activator IL-16 in the peripheral blood and found that IL-2 concentrations were increased in the peripheral blood of patients with stage IV colorectal cancer (one-way ANOVA, IL-2 control vs stage IV, *p* = 0.0226) (**Figure 5A**). Natural killer (NK) cells and Th1 cells share CXCR3 receptors and exert anti-tumor effects in response to stimulation by the specific ligands CXCL9, CXCL10, and CXCL11. Next, we analyzed the distribution of NK cell-related cytokines, including CXCR3 and CX3CR1 ligands, in the peripheral blood of the participants. The concentrations of CXCL9, CXCL10, and CXCL11 in the peripheral blood of patients with stage IV colorectal cancer increased (one-way ANOVA, CXCL9 control vs stage IV, *p* = 0.0010; CXCL10 stage I vs stage IV, *p* = 0.0478; CXCL11 stage IIIA vs stage IV, *p* = 0.0190), while the concentrations of CX3CL1 in the peripheral blood of patients with stage II, IIIB-C, and IV colorectal cancer were decreased (one-way ANOVA, CX3CL1 stage I vs stage II, *p* = 0.0015, stage I vs stage IIIB-C, *p* = 0.0048, stage I vs stage IV, *p* = 0.0328), suggesting the phenomenon of differential recruitment to NK cells in different colorectal cancer patients, and that in stage II, IIIB-C, and some stage IV patients may have suppressed the anti-tumor function of NK cells, while some stage IV patients had activated anti-tumor function of NK cells (**Figure 5B**). Subsequently, we analyzed the distribution of Th1 cell-related cytokines in the peripheral blood of participants, and found that the CXCR6 ligand CXCL16 was slightly upregulated in the peripheral blood of stage IV colorectal cancer patients; however, no significant differences were observed (**Figure 5C**). In contrast to Th1 cells, Th2 cells express CCR3, CCR4, and CCR8 receptors on their surface and can promote tumor progression by exerting immunomodulatory functions in response to stimulation by the corresponding ligands. The concentrations of CCL1, CCL11, and CCL26 were elevated in the peripheral blood of patients with stage IV colorectal cancer (one-way ANOVA, CCL1 control vs stage IV, *p* < 0.0001; CCL11 control vs stage IV, *p* < 0.0001; CCL26 control vs stage IV, *p* < 0.0001), suggesting that activation of the pro-tumor function of Th2 cells may exist in stage IV patients (**Figure 5D**). In addition, we detected upregulation of CCL25 and CCL27 in the peripheral blood of patients with stage IV colorectal cancer (one-way ANOVA, CCL25 control vs stage IV, *p* = 0.0100; CCL27 control vs stage IV, *p* = 0.0015), which were associated with the recruitment of type II intrinsic lymphocytes (ILC2s) and regulatory T cells *in vivo*, respectively (**Figures 5E, F**).

### 3.6 Relationship between distribution of peripheral blood cytokines and lymph node metastasis or primary tumor size in CRC patients

**Figure 6:**
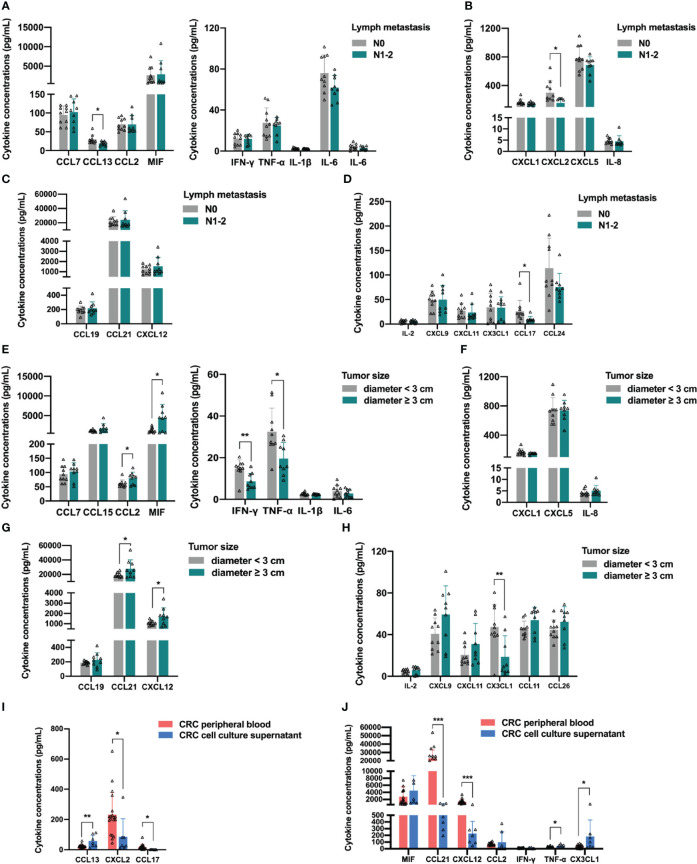
Relationship between distribution of peripheral blood cytokines and lymph node metastasis or primary tumor size in colorectal cancer patients. **(A)** Analysis of the monocyte-macrophage system associated cytokines. The concentration of monocyte chemotactic protein CCL13 was lower in the N1-2 group than in the N0 group. **(B)** Analysis of the neutrophil-associated cytokines. The concentration of CXCL2 was lower in the N1-2 group than in the N0 group. **(C)** The analysis of dendritic cell-related cytokines showed no significant differences between the two groups. **(D)** Analysis of the lymphocyte-associated cytokines. The concentration of CCL17 was lower in the N1-2 group than in the N0 group. **(E)** Analysis of the monocyte-macrophage system associated cytokines. CCL2 and MIF were significantly upregulated in the tumor diameter ≥3 cm group, and IFN-γ and TNF-α were significantly downregulated in the tumor diameter ≥ 3 cm group. **(F)** The analysis of the neutrophil-associated cytokines showed no significant differences between the two groups. **(G)** Analysis of the antigen-presenting cells-related cytokines. CCL21 and CXCL12 were significantly upregulated in the tumor diameter ≥3 cm group. **(H)** Analysis of the lymphocyte-associated cytokines. CX3CL1 was significantly downregulated in the tumor diameter ≥3 cm group. **(I, J)** Analysis of the differential cytokines. The concentration of CXCL2, CCL17, CCL21 and CXCL12 were significantly lower in CRC cell line culture supernatant than in CRC patients’ peripheral blood. Data are presented as mean ± SD, Student’s t-test, *p < 0.05, **p < 0.01, ***p < 0.001.

In a previous analysis, we found that the differential upregulation of cytokines in peripheral blood was mainly concentrated in stage IV colorectal cancer patients with distant metastasis. Therefore, we analyzed whether the distribution of cytokines in the peripheral blood was related to lymph node metastasis in colorectal cancer. We excluded data from normal controls and stage IV colorectal cancer patients, divided the remaining participants into N0 and N1-2 groups according to the presence of lymph node metastasis, and compared the levels of each cytokine in the peripheral blood of patients in the two groups. In addition, we performed a more detailed analysis of the clinical data of CRC patients between these groups (**Table S3**), and there were no significant differences in age, sex, BMI category, tumor location, tumor histology, and tumor size. **Figure 6A** shows the analysis of the monocyte-macrophage system-associated cytokines in the two groups, in which only the monocyte chemotactic protein CCL13 was lower in the N1-2 group than in the N0 group (Student’s t-test, *p* = 0.0200), and no significant differences were observed between the two groups in the levels of the remaining monocyte/macrophage chemotaxis-related proteins and macrophage polarization-related factors. According to previous reports, CCL13 is important in the recruitment of monocytes and eosinophils (20, 21), and is positively associated with better prognosis in colorectal, breast, and ovarian cancer (22). Among the neutrophil-associated cytokines, only CXCL2 was reduced in the N1-2 group (Student’s t-test, *p* = 0.0310), and no significant differences were observed for the remaining cytokines (**Figure 6B**). CXCL2 is a tumor-promoting factor that recruits neutrophils to the TME to exert an immunosuppressive effect (23, 24). Analysis of DC-related cytokines showed no significant differences between the N0 and N1-2 groups (**Figure 6C**). In the analysis of lymphocyte-associated cytokines, only CCL17 concentration was reduced in the N1-2 group (Student’s t-test, *p* = 0.0397) (**Figure 6D**). CCL17 is a chemotactic factor for Th2 and Treg cells owing to the expression of CCR4 in these cells (25); however, it also exerts an anti-cancer effect by causing the infiltration of TIL into the tumor (26). Increased expression of CCL17 improves prognosis in lung, colorectal, and breast cancer (22). In this part of the analysis, no significant upregulation of any cytokine was observed in colorectal cancer patients with lymph node metastasis.

The levels of cytokines in the peripheral blood of patients with tumors are often related to tumor burden, which is the size of the primary tumor. Therefore, we analyzed whether the distribution of cytokines in the peripheral blood was related to the size of the primary tumor in colorectal cancer patients. We excluded data from normal participants and stage IV colorectal patients, divided the remaining participants into a tumor diameter <3 cm group and a tumor diameter ≥3 cm group according to the size of the primary tumor, and then compared the levels of each cytokine in the peripheral blood of the two groups. In addition, we performed a more detailed analysis of the clinical data of CRC patients between the two groups (**Table S4**), and there were no significant differences in age, sex, BMI category, tumor location, tumor histology, and lymph metastasis between the two groups. As expected, the analysis of monocyte-macrophage system-associated cytokines showed that macrophage chemotaxis-related protein CCL2 and MIF were upregulated in the tumor diameter ≥3 cm group (Student’s t-test, CCL2, *p* = 0.0285; MIF, *p* = 0.0105), whereas the M1 polarization-related factors IFN-γ and TNF-α were downregulated in the tumor diameter ≥3 cm group (Student’s t-test, IFN-γ, *p* = 0.0041; TNF-α, *p* = 0.0104), indicating that macrophages were recruited more; conversely, the polarization of anti-tumor M1 macrophages was restricted (**Figure 6E**). In contrast, no significant difference was found between the two groups in the levels of neutrophil-associated growth factors, suggesting that the size of the primary tumor had little effect on neutrophil recruitment and activation (**Figure 6F**). In addition, the levels of DC-associated cytokine CCL21 and B cell-associated cytokine CXCL12 were upregulated in the tumor diameter ≥3 cm group (Student’s t-test, CCL21, *p* = 0.0471; CXCL12, *p* = 0.0432), suggesting that as the primary colorectal cancer lesion grows, the tumor secretes more cytokines, such as CCL21 and CXCL12, into the peripheral blood to recruit APCs (**Figure 6G**). Finally, analysis of lymphocyte-associated factors showed that the NK cell-associated cytokine CX3CL1 was downregulated in the tumor diameter ≥3 cm group (Student’s t-test, *p* = 0.0086). The results of the analysis of stage IV patients in the previous section strongly suggest the existence of an immune escape mechanism that inhibits NK cell recruitment and anti-tumor function by reducing the level of CX3CL1 in peripheral blood during the progression of colorectal cancer (**Figure 6H**). These results indicate that the differential upregulation of cytokines in peripheral blood was mainly concentrated in CRC patients with distant metastasis and related to the size of the primary tumor. Hence, we next explored the sources of differential cytokines by comparing the concentrations of differential cytokines in the peripheral blood within the CRC cell line culture supernatant. The levels of CCL13, MIF, CCL2, IFN-γ, TNF-α, and CX3CL1 were not significantly different between the two groups, indicating that these cytokines were mainly secreted by CRC cells. The concentrations of CXCL2, CCL17, CCL21, and CXCL12 in the CRC cell line culture supernatant were lower than those in patients’ peripheral blood (Student’s t-test, CXCL2, *p* = 0.0271; CCL17, *p* = 0.0283; CCL21, *p* < 0.0001; CXCL12, *p* = 0.0002), indicating that these cytokines were partially secreted by CRC cells (**Figures 6I, J**).

### 3.7 Role of chemokines and their receptors in co-localization of immune cells in TME

**Figure 7:**
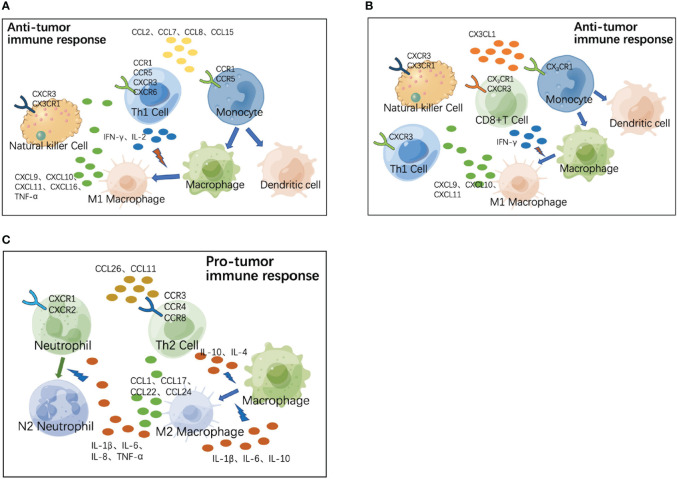
Role of chemokines and their receptors in co-localization of immune cells in tumor microenvironment (TME). **(A)** During the progression of colorectal cancer, CCR1 and CCR5 ligands (CCL2, CCL7, CCL8, CCL15, and so on) recruit monocytes and Th1 cells to the TME, where monocytes are precursors of macrophages and dendritic cells (DCs) and play the role of antigen presentation. Th1 produces IFN-γ and IL-2, and IFN-γ induces M1 polarization of macrophages. M1 macrophages exhibit enhanced antigen-presenting activity, while secreting CXCR3 ligands (CXCL9, CXCL10, CXCL11, and CXCL16) to recruit Th1 and natural killer (NK) cells and secrete TNF-α; synergistically exerting anti-tumor functions. **(B)** In the early stages of colorectal cancer, the CX3CR1 ligand (CX3CL1) recruits NK cells, monocytes, and activated T cells to the TME. Monocytes differentiate into macrophages and DCs to play the role of antigen presentation. Activated T cells secrete IFN-γ, upregulating the expression of CXCR3 ligands (CXCL9, CXCL10, and CXCL11) and recruiting Th1 and NK cells to perform anti-tumor functions. **(C)** During tumor progression, Th2 cells are recruited to the TME by CCR3 ligands (CCL26, CCL11, and so on), and produce IL-4 and IL-10, which induce M2 polarization of macrophages. M2 macrophages secrete CCL1, CCL17, CCL22, CCL24, and so on to recruit Th2 cells, as well as secreting inflammatory cytokines (IL-1β, IL-6, IL-10, and so on) to continuously induce M2 polarization of macrophages. M2 macrophages secrete inflammatory cytokines (TNF-a, IL-1β, and so on) to directly recruit neutrophils, while inflammation enhances endothelial adhesion and migration of neutrophils. Inflammatory cytokines (IL-8, IL-6, and so on) in TME can induce M2 polarization of neutrophil cells; synergistically exerting pro-tumor functions.

Because different types of immune cells may share the same chemokine receptors, they can co-localize in response to the same chemokines and exert synergistic effects in tissues. We next attempted to analyze cytokines and chemokines with significantly different distributions in the peripheral blood of colorectal cancer patients to determine their role in the co-localization of immune cells in the TME. As shown in **Figure 7A**, both Th1 cells and monocytes commonly express CCR1 and CCR5 receptors, whereas both Th1 and NK cells express CXCR3 receptors on their surface. During the progression of colorectal cancer, CCR1 and CCR5 ligands (CCL2, CCL7, CCL8, CCL15, and so on) recruit monocytes and Th1 cells to the TME, where monocytes are precursors of macrophages and DCs and play a role in antigen presentation; Th1 produces IFN-γ and IL-2, and IFN-γ induces M1 polarization of macrophages. M1 macrophages exhibit enhanced antigen-presenting activity while secreting CXCR3 ligands (CXCL9, CXCL10, CXCL11) and CXCL16 to recruit Th1 and NK cells and secrete TNF-α, synergistically exerting anti-tumor functions. **Figure 7B** shows another anti-tumor mechanism in the early stages of colorectal cancer. NK cells, monocytes, and some activated T cells commonly express CX3CR1 receptors, whereas both Th1 and NK cells express CX3CR3, a homologous receptor of CX3CR1 on the surface. The CX3CR1 ligand (CX3CL1) recruits NK cells, monocytes, and activated T cells to the TME; monocytes differentiate into macrophages and DCs to play a role in antigen presentation; activated T cells secrete IFN-γ, upregulating the expression of CXCR3 ligands (CXCL9, CXCL10, and CXCL11) to recruit Th1 and NK cells, synergistically exerting anti-tumor functions. As the tumor progresses, the immune response gradually shifts from anti-tumor to pro-tumor mode (**Figure 7C**). During tumor progression, Th2 cells are recruited to the TME by CCR3 ligands (CCL26, CCL11, and so on), and produce IL-4 and IL-10, which induce M2 polarization of macrophages. M2 macrophages secrete CCL1, CCL17, CCL22, and CCL24 to recruit Th2 cells, as well as inflammatory cytokines (IL-1β, IL-6, IL-10, and so on) to continuously induce M2 polarization of macrophages. M2 macrophages secrete inflammatory cytokines (TNF-α, IL-1β, and so on) to directly recruit neutrophils, while inflammation enhances endothelial adhesion and migration of neutrophils. Inflammatory cytokines (IL-8, IL-6, and so on) in the TME can induce M2 polarization of neutrophil cells and synergistically exert pro-tumor functions.

## 4 Discussion

CRC is one of the most common malignant tumors of the digestive tract and the third most common cancer in the world (27). Inflammation plays an important role in all stages of tumor development (28). Moreover, chronic inflammation is associated with an increased risk of cancer, and the presence of an inflammatory TME has been proposed as an important marker of cancer (29). Inflammatory factors play a key role in the occurrence and development of tumors, either directly acting on tumor cells (i.e. induction of epithelial mesenchymal transformation and activation of cancer stem cells) or indirectly playing a multi-effect role by promoting favorable conditions in the microenvironment (including inhibiting the anti-tumor activity of immune cells), ultimately promoting the survival, proliferation, invasion and metastasis of cancer cells (19, 30).

Cytokines are a class of small molecular proteins or peptides with biological activity and can be divided into pro-inflammatory and anti-inflammatory cytokines (31). The pro-inflammatory cytokines mainly include TNFs, IL-1 family members, IL-2, IL-6, IL-8, IL-12, IFNs, and MIF. The anti-inflammatory cytokines included IL-4, IL-13, and IL-10 family members, the transforming growth factor-β (TGF-β) family, and soluble receptors of pro-inflammatory cytokines. In addition, the chemokine (CXC, CC, XC, and CX3C) family and hematopoietic growth factors (GM-CSF) are also included (19, 32).

In this comprehensive investigation of circulating cytokines and colorectal cancer, our main observation was that 17 markers involved in several components of the inflammatory process, CCL7, CCL8, CCL15, CCL2, MIF, IL-1β, CXCL2, CXCL5, CXCL6, IL-8, CCL19, CCL21, CXCL9, CXCL10, CCL11, CCL26, and IL-2, were significantly upregulated in the peripheral blood of patients with advanced colorectal cancer. In addition, six homeostatic chemokines, CXCL12, CCL1, CCL20, CCL25, CCL27, and CXCL11, were upregulated in the peripheral blood of patients with advanced colorectal cancer, which has also been reported to be associated with tumor progression (33–35). In particular, the concentrations of IFN-γ and CX3CL1 were significantly downregulated in the peripheral blood of patients with advanced colorectal cancer, which may be associated with an anti-tumor immune response (36–38).

The immunologic TME plays a vital role in the development of CRC, where resident cells communicate with neighboring cells through soluble factors, such as cytokines or chemokines, to produce a pro-tumor or anti-tumor immune response (39). Our results are consistent with those of previous studies that reported elevated levels of several circulating cytokines in cancer patients, namely increased concentrations of cytokines MIF, TNF-α, IL-6, IL-8, IL-17, IL-23, IL-10, and TGF-β in the peripheral blood of cancer patients, leading to simultaneous immune activation and immunosuppression in cancer patients (40, 41). Previous studies have reported elevated levels of several circulating cytokines in colorectal cancer patients, including IL-1β, IL-8, and TNF-α, which are thought to promote tumor development in the context of chronic inflammation (19). However, studies have also reported higher levels of IFN-γ in the peripheral blood of patients with colorectal cancer, which inhibits tumor formation and protects the host from tumor formation (42). Thus, the activity of some anti-tumor immune factors may be counteracted in the presence of some contrary signals, such as those imposed by other pro-tumor cytokines found in patients.

In this study, we propose possible patterns of anti-tumor immune activation and immunosuppression in the colorectal cancer microenvironment and propose key cytokines in each pattern based on our main observations. High concentrations of CX3CL1 exist in the immune microenvironment of early colorectal cancer, which recruits NK cells, monocytes, and activated T cells to the tumor region; monocytes differentiate into macrophages and DCs for antigen presentation; activated T cells secrete IFN-γ, upregulating the expression of CXCR3 ligands to recruit Th1 and NK cells, promoting the activation of CD8+T cells and its killing effect on tumor cells. With the progression of tumor, chemokines such as CCL2, CCL7, CCL8, and CCL15 in the microenvironment of colorectal cancer gradually increase, recruiting monocytes and Th1 cells to the tumor region. Th1 cells produce IFN-γ to induce M1-polarization of macrophages, and produce IL-2 to enhance the function of T cells; M1 macrophages recruit Th1 cells and NK cells for synergistic anti-tumor function. With further growth of the tumor, the immune response gradually changed from anti-tumor to pro-tumor mode, and the high concentration of CCL26 and CCL11 in the TME recruits Th2 cells to the tumor area. Th2 cells produce IL-4 and IL-10 to induce M2 polarization of macrophages; M2 macrophages secrete CCLs to recruit Th2 cells, while secreting IL-1β, IL-6, and IL-10 to induce M2 polarization of macrophages continuously; TNF-α and IL-1β secreted by M2 macrophages directly recruit neutrophils, and inflammation enhances the endothelial adhesion and migration of neutrophils; IL-8 and IL-6 induce the M2 polarization of neutrophils to play a synergistic role in promoting tumor development.

Many studies have used the Luminex assay to detect circulating cytokines in tumors. Most of these studies have focused on the development of biomarkers for early tumor screening and risk predictive cytokines (43, 44). For instance, Luminex’s liquid array-based multiple immunoassay was used in aggressive prostate cancer to screen serum cytokines and identify less invasive and easily applicable serum cytokine-derived biomarkers. Serum TRAIL and IL-10 were identified as new biomarkers for prostate cancer detection and risk stratification (45). In addition, some articles have reported the characterization of soluble molecules in malignant tumors (46). Sirven et al. provided the first breast tumor-specific classifier computed on breast tissue-derived secretome data (47). Similar to the purpose of the latter type of study, our study aimed to analyze the secretome data of colorectal cancer to characterize the relationship between circulatory cytokines, tumor cells, and the microenvironment, which has been relatively limited in previous studies. The TME consists of tumor cells, mesenchymal cells, endothelial cells, immune cells, and their secretions. In this microenvironment, various cell types interact with each other and regulate tumor growth, progression, and metastasis. Inflammation may precede the development of malignancies, and in other cases, oncogenic changes in malignant cells lead to the formation of an inflammatory environment that promotes tumor development (4). The inflammatory environment contributes to the proliferation and survival of malignant cells, stimulates angiogenesis and metastasis, disrupts adaptive immunity, and alters the response to tumor therapy. Previous studies have proposed that most of the elevated circulating factors found in CRC patients are considered pro-inflammatory mediators, suggesting that inflammation plays an important role in the microenvironment of colorectal cancer and is a major source of circulating pro-inflammatory factors, possibly involving tumor cells as well as non-malignant stromal cells and inflammatory cells (48). In our study, we found that the upregulated cytokines in the peripheral blood of most patients with colorectal cancer were positively correlated with tumor burden. This finding is consistent with previous reports that cancer patients exhibit an inflammatory state caused and maintained by tumor volume (19).

This study has some limitations. For instance, although the association of inflammatory cytokines with colorectal cancer is biologically plausible, there may be some margin of error in our observations due to the large number of cytokines evaluated. In total, we identified 25 circulating cytokines that were statistically significant in patients with colorectal cancer; however, repeated validation and laboratory studies are needed before we can determine the specific function and clinical translational value of these results. At the same time, owing to the low detectability or poor reproducibility (large differences within groups) of some cytokines, our experimental results may have a certain degree of chance. In addition, cytokine levels were measured at only one point in time for each participant. Therefore, we may have overlooked how they might change over time.

In conclusion, our study extends the observation of peripheral blood cytokine distribution in patients with colorectal and provides laboratory evidence for the association of pro-inflammatory and anti-inflammatory cytokines, chemokines, and growth factors with the development and progression of colorectal cancer. Moreover, we propose that different types of immune cells may share the same chemokine receptor, respond to the same chemokines, and co-locate in the TME to perform synergistic pro-tumor or anti-tumor functions. Chemokines and cytokines affect tumor metastasis and prognosis and may be potential therapeutic targets.

## Data availability statement

The original contributions presented in the study are included in the article/**Supplementary Material**. Further inquiries can be directed to the corresponding authors.

## Author contributions

All authors meet the authorship requirements. Conception and design: WL, FC, and AL. Development of methodology: HG, FC, and ZW. Acquisition of data (provided animals, acquired and managed patients, provided facilities, etc.): ZL, ZQX, SW, and WF. Analysis and interpretation of data (e.g., statistical analysis, biostatistics, computational analysis): WL, HG, ZL, and YZ. Writing, review, and/or revision of the manuscript: WL, ZFX, and AL. Administrative, technical, or material support (i.e., reporting or organizing data, constructing databases): JH, WL, and YCZ. Study supervision: XS, JZ, and ZW. Revision director: YPZ and XS. All authors contributed to the article and approved the submitted version.
