# Supplementary material for: Cytokine concentration in peripheral blood of patients with colorectal cancer
Source: Front Immunol. 2023 Mar 30;14:1175513. doi: 10.3389/fimmu.2023.1175513 (PMC10098211; doi:10.3389/fimmu.2023.1175513)
Supplement: Supplementary file 1 [file Table_1.docx]

**Table S1 Clinical characteristics for Luminex assay.**

| Sample ID | Patient sex | Patient age | Tumor location | TNM stage |
| --- | --- | --- | --- | --- |
| C1 | 47 | F | / | / |
| C2 | 64 | M | / | / |
| C3 | 51 | M | / | / |
| C4 | 71 | M | / | / |
| C5 | 58 | M | R | I |
| C6 | 78 | F | R | I |
| C7 | 75 | F | S | I |
| C8 | 77 | M | S | I |
| C9 | 66 | F | T | I |
| C10 | 45 | F | R | I |
| C11 | 67 | M | A | II |
| C12 | 68 | F | S | II |
| C13 | 73 | M | R | II |
| C14 | 66 | M | A | II |
| C15 | 43 | M | S | IIIA |
| C16 | 46 | F | R | IIIA |
| C17 | 64 | F | R | IIIA |
| C18 | 61 | M | A | IIIA |
| C19 | 63 | M | A | IIIA |
| C20 | 66 | M | S | IIIA |
| C21 | 68 | M | S | IIIB |
| C22 | 63 | M | R | IIIC |
| C23 | 84 | F | T | IIIB |
| C24 | 66 | M | A | IV |
| C25 | 63 | M | S | IV |
| C26 | 57 | M | T | IV |

Tumor location: A, ascending colon; T, transverse colon; D, descending colon; S, sigmoid colon, R, rectum.

**Table S2 Additional information of cytokines for Luminex assay.**

| Cytokines | Name in Bio-Plex Pro Human Chemokine Panel | Description |
| --- | --- | --- |
| CCL21 | 6Ckine/CCL21.(12) | C-C Motif Chemokine Ligand 21; Secondary Lymphoid Tissue Chemokine |
| CXCL5 | ENA-78/CXCL5.(73) | C-X-C Motif Chemokine Ligand 5; Epithelial-Derived Neutrophil-Activating Protein 78 |
| CCL22 | MDC/CCL22.(29) | Macrophage-Derived Chemokine; C-C Motif Chemokine 22 |
| MIF | MIF.(35) | Macrophage Migration Inhibitory Factor |
| CCL15 | MIP-1delta/CCL15.(66) | C-C Motif Chemokine Ligand 15; Macrophage Inflammatory Protein 1delta |
| CXCL12 | SDF-1a+b/CXCL12.(22) | C-X-C Motif Chemokine Ligand 12;  Stromal Cell-Derived Factor 1 |
| CCL25 | TECK/CCL25.(46) | C-C Motif Chemokine Ligand 25; Thymus Expressed Chemokine |
| CCL27 | CTACK/CCL27.(72) | C-C Motif Chemokine Ligand 27; Cutaneous T-Cell Attracting Chemokine |
| CCL24 | Eotaxin-2/CCL24.(30) | C-C Motif Chemokine Ligand 24; Myeloid Progenitor Inhibitory Factor 2 |
| CXCL1 | Gro-a/CXCL1.(61) | C-X-C Motif Chemokine Ligand 1; Growth-Regulated Alpha Protein |
| CXCL2 | Gro-b/CXCL2.(78) | C-X-C Motif Chemokine 2; Growth-Regulated Protein Beta |
| IL-16 | IL-16.(27) | Interleukin 16; Lymphocyte Chemoattractant Factor |
| IL-4 | IL-4.(52) | Interleukin 4 |
| CXCL10 | IP-10/CXCL10.(48) | C-X-C Motif Chemokine Ligand 10; Protein 10 From Interferon (Gamma) |
| CCL2 | MCP-1/CCL2.(53) | C-C Motif Chemokine Ligand 2; Monocyte Chemoattractant Protein-1 |
| CCL7 | MCP-3/CCL7.(26) | C-C Motif Chemokine Ligand 7; Monocyte Chemoattractant Protein 3 |
| CCL19 | MIP-3b/CCL19.(76) | C-C Motif Chemokine Ligand 19; Macrophage Inflammatory Protein 3 Beta |
| CCL23 | MPIF-1/CCL23.(37) | C-C Motif Chemokine Ligand 23; Myeloid Progenitor Inhibitory Factor 1 |
| CXCL16 | SCYB16/CXCL16.(64) | C-X-C Motif Chemokine Ligand 16; Scavenger Receptor For Phosphatidylserine |
| CXCL13 | BCA-1/CXCL13.(74) | C-X-C Motif Chemokine Ligand 13; B-Cell-Attracting Chemokine 1 |
| CCL26 | Eotaxin-3/CCL26.(65) | C-C Motif Chemokine Ligand 26; Macrophage Inflammatory Protein 4 |
| CCL11 | Eotaxin/CCL11.(43) | C-C Motif Chemokine Ligand 11; |
| CX3CL1 | Fractalkine/CX3CL1.(77) | C-X3-C Motif Chemokine Ligand 1; |
| CXCL6 | GCP-2/CXCL6.(15) | C-X-C Motif Chemokine Ligand 6; Granulocyte Chemotactic Protein 2 |
| GM-CSF | GM-CSF.(34) | Granulocyte-Macrophage Colony-Stimulating Factor |
| CCL1 | I-309/CCL1.(20) | C-C Motif Chemokine Ligand 1; T Lymphocyte-Secreted Protein I-309 |
| CXCL11 | I-TAC/CXCL11.(25) | C-X-C Motif Chemokine Ligand 11; Interferon-Inducible T-Cell Alpha Chemoattractant |
| IFN-g | IFN-g.(21) | Interferon Gamma |
| IL-10 | IL-10.(56) | Interleukin 10 |
| IL-1b | IL-1b.(39) | Interleukin 1 Beta |
| IL-2 | IL-2.(38) | Interleukin 2; T Cell Growth Factor |
| IL-6 | IL-6.(19) | Interleukin 6 |
| IL-8 | IL-8/CXCL8.(54) | C-X-C Motif Chemokine Ligand 8; Interleukin 8 |
| CCL8 | MCP-2/CCL8.(57) | C-C Motif Chemokine Ligand 8; Monocyte Chemoattractant Protein 2 |
| CCL13 | MCP-4/CCL13.(28) | C-C Motif Chemokine Ligand 13; Monocyte Chemoattractant Protein 4 |
| CXCL9 | MIG/CXCL9.(14) | C-X-C Motif Chemokine Ligand 9; Monokine Induced By Gamma Interferon |
| CCL3 | MIP-1a/CCL3.(55) | C-C Motif Chemokine Ligand 3; Macrophage Inflammatory Protein 1-Alpha |
| CCL20 | MIP-3a/CCL20.(62) | C-C Motif Chemokine Ligand 20; Macrophage Inflammatory Protein 3 Alpha |
| CCL17 | TARC/CCL17.(67) | C-C Motif Chemokine Ligand 17; Thymus And Activation-Regulated Chemokine |
| TNF-a | TNF-a.(36) | Tumor Necrosis Factor-Alpha |

**Table S3 Additional clinical information for Luminex assay.**

| Clinicopathologic parameters | Case | Lymph metastasis | | P- value |
| --- | --- | --- | --- | --- |
|  |  | N0 | N1-2 |  |
| Total | 19 | 10 | 9 |  |
| Age |  |  |  | 0.5820 |
| <50 | 3 | 1 | 2 |  |
| ≥50 | 16 | 9 | 7 |  |
| Gender |  |  |  | 0.6499 |
| Male | 11 | 5 | 6 |  |
| Female | 8 | 5 | 3 |  |
| Histology |  |  |  | >0.9999 |
| Low | 2 | 1 | 1 |  |
| Moderate + High | 17 | 9 | 8 |  |
| BMI category (Mean ± SD) |  | 25.47 ± 3.530 | 25.18 ± 3.633 | 0.8610 |
| Tumor location |  |  |  | >0.9999 |
| Right | 6 | 3 | 3 |  |
| Left | 13 | 7 | 6 |  |
| Tumor size |  |  |  | >0.9999 |
| ≥3 cm | 9 | 5 | 4 |  |
| <3 cm | 10 | 5 | 5 |  |
| T class (Invasion depth) |  |  |  | >0.9999 |
| T1-2 | 12 | 6 | 6 |  |
| T3-4 | 7 | 4 | 3 |  |

**Table S4 Additional clinical information for Luminex assay.**

| Clinicopathologic parameters | Case | Tumor size | | P- value |
| --- | --- | --- | --- | --- |
|  |  | diameter <3 cm | diameter≥3 cm |  |
| Total | 19 | 10 | 9 |  |
| Age |  |  |  | >0.9999 |
| <50 | 3 | 2 | 1 |  |
| ≥50 | 16 | 8 | 8 |  |
| Gender |  |  |  | >0.9999 |
| Male | 11 | 6 | 5 |  |
| Female | 8 | 4 | 4 |  |
| Histology |  |  |  | >0.9999 |
| Low | 2 | 1 | 1 |  |
| Moderate + High | 17 | 9 | 8 |  |
| BMI category (Mean ± SD) |  | 24.29 ± 4.047 | 26.49 ± 2.445 | 0.1759 |
| Tumor location |  |  |  | 0.6285 |
| Right | 6 | 4 | 2 |  |
| Left | 13 | 6 | 7 |  |
| T class (Invasion depth) |  |  |  | 0.0198 |
| T1-2 | 12 | 9 | 3 |  |
| T3-4 | 7 | 1 | 6 |  |
| N class (Lymph metastasis) |  |  |  | >0.9999 |
| N0 | 10 | 5 | 5 |  |
| N1-2 | 9 | 5 | 4 |  |
